# How behavioural changes can help deal with the health impacts of climate change to achieve SDG 13 (climate action)

**DOI:** 10.1111/aphw.70221

**Published:** 2026-09-15

**Authors:** Walter Leal Filho, Franziska Wolf

**Affiliations:** ^1^ Hamburg University of Applied Sciences Research and Transfer Centre “Sustainable Development and Climate Change Management” Hamburg Germany; ^2^ WSB Merito University Wroclaw Poland; ^3^ Department of Natural Sciences Manchester Metropolitan University Manchester UK

**Keywords:** action, climate change, health‐ impacts, SDG 13, SDG 3

## Abstract

Climate change is a major public health issue in the 21st century, and the links between climate change and health are increasingly becoming recognised by researchers and policy‐makers. Interventions aimed at promoting sustainable behaviours, that is, reducing harmful activities and inducing pro‐environmental action as well as adaptive capacities, can be diverse in targeting changes in culture, feedback loops and technological development. A focus on changing both individual and collective behaviours is indispensable to reduce the negative impacts on health. This overview outlines the connections between climate change, health and behavioural change and discusses how such changes can assist in efforts towards handling the impacts of climate change. This is of particular relevance as it relates to achieving the Sustainable Development Goal (SDG) 13 (Climate Action) and, simultaneously, contribute also to SDG 3 (Good Health and Well‐being). Focusing on the health benefits of pro‐environmental action today may pave the way for improvements. A holistic climate change response, however, needs to consider health concerns from a multisectoral perspective and engage stakeholders at all levels—from neighbourhood communities to international structures as health challenges linked to climate change intensify.

## THE LINKS BETWEEN CLIMATE CHANGE AND HEALTH

It is generally agreed that climate change is among the most pressing public health challenges of the 21st century and will influence human health in multiple spheres (Campbell‐Lendrum et al., 2023; Rocque et al., 2021). The Intergovernmental Panel on Climate Change (IPCC) has highlighted the consequences on health from global climate change (Cissé et al., 2022). And these implications include a surge in deaths from heat exposure, outbreaks of infectious diseases in the wake of severe weather events and significant health repercussions from forced migration because of the increase in sea level, rising storms and storm surges. Particularly vulnerable groups such as the elderly and the poor are among the most at‐risk, and they are facing more acute threats from rising temperatures, that is, heat‐related mortality and infections with waterborne pathogens. Furthermore, climate change promotes transmission of vector‐borne diseases to new environments, which also endangers both human and animal health, while its control and prevention have become a core issue of public and environmental health (Savic et al., 2019).

The predominance of negative impacts emphasises the urgent need for coordinated adaptation strategies between the health sector and other sectors, especially agriculture and water (Bowen & Ebi, 2015), although health effects of climate change might also show certain benefits, for example, suggesting somewhat reduced cold wave related mortality in distinct regions (Wang et al., 2016) or referring to some shorter viral latency periods (Wieczynski et al., 2023). With increasing global temperatures and climate‐related changes, a link between environmental change and public health becomes even more evident. Increases in diseases such as respiratory illnesses, vector‐borne diseases and more emphasise the need for novel interventions to encourage personal as well as environmental health beneficial behaviours (Abbass et al., 2022).

The link between climate change and health is also defined as multifactorial, with both direct and indirect effects. For example, increasing average temperatures and more frequent extreme weather events can diminish levels of physical activity and sleep quality for people, while exacerbating symptoms of mental health from the stress and trauma that accompany climate‐related disasters. Adopting the Planetary Health concept, coined in 2013 by the Rockefeller foundation, further supports this worldview by framing it in a wholistic way, noting that human health is related directly to the health of ecological systems. As such, crossing vital planetary thresholds, particularly those related to climate change, can threaten global public health and human well‐being, while those alterations also include environmental, and especially climatic, perturbations that impose significant human health burdens (Whitmee et al., 2015).

Equally, food insecurity and malnutrition exacerbated by climate change continue to pose significant challenges, further complicating food choices and overall health (Edmondson et al., 2022; Zyoud & Zyoud, 2024). The intricate dynamics of climate change and health make it difficult to draw a distinction between environmental and biological influences on health. Environmental and biological influences also lead to uncertainties in how much the two interact (McMichael et al., 1996).

Given the changing environment, building and continuously refining a robust scientific evidence base is essential to better understand the unique role climate change plays in human health. As a result, climate impacts are becoming an increasingly critical focus for both researchers and policy‐makers. Strengthening this knowledge base supports efforts to promote societal resilience, helping communities better withstand the adverse environmental conditions in which they live.

In addition, behavioural change can play a key role in addressing the health impacts associated with climate change, particularly within the SDG 13framework. SDG13 fosters ‘urgent action to combat climate change and its impacts’. It aims at strengthening resilience to climate hazards, integrating climate measures into policy and improving education on mitigation (UN, 2025). For example, adopting plant‐based diets (Viroli et al., 2023) and actively working to minimise food waste (Mariam et al., 2020) not only reduces the individual carbon footprint but also contributes to improving overall health, illustrating the dual benefits of such lifestyle modifications. Such health co‐benefit approaches align perfectly with the goals of SDG 13, which advocates integrative measures that also encompass both health and climate action (Edmondson et al., 2022).

## THE CONNECTIONS BETWEEN CLIMATE CHANGE, HEALTH COMMUNICATIONS AND BEHAVIOURAL CHANGE

Research shows that discussing the health consequences of climate change and highlighting the benefits of solutions can increase public awareness and engagement (Campbell et al., 2023; Thier et al., 2025). Framing messages around individual health, such as the benefits of reduced air pollution through replacing fossil energy sources with renewable energy, makes them more understandable and acceptable (Peters et al., 2022). An impactful call to action that aligns with community values can enhance the effectiveness of these messages, demonstrating how broad support can involve the community. Using language that emphasises the direct health advantages can encourage individuals to embrace eco‐friendly actions, instead of merely concentrating on the long‐term effects.

Generally, addressing climate change effectively requires behavioural change, either collective or individual‐level changes based on both conscious and unconscious action, to achieve a significant societal transformation (van Valkengoed et al., 2022). Whitmarsh et al. (2021) recommend (a) emphasising high‐impact behaviours in the areas of, for example, mobility, food, consumption or resilience and targeting high‐emitting groups; (b) designing interdisciplinary interventions to act on multiple drivers, barriers and consider the overall behavioural context; and (c) using windows of opportunity to counter negative habits.

Concerning climate‐friendly, that is, emission‐reduction related, behaviours imperative to slow down climate change, Guan et al. (2025) refer to already impactful and broadly available approaches, for example, reducing consumption of animal‐based food, sugar and unhealthy processed food products. However, they state that for effective Greenhouse gas (GHG) reduction, one has to consider sources and opportunities to change, and one needs to take direct and indirect rebound effects into account as well when developing behavioural interventions. A direct rebound effect refers to unintended negative consequences of a seemingly positive action, for example, when improved energy efficiency results in cheaper energy services, which in turn may trigger their increased consumption. An indirect rebound effect can be considered as linked to energy efficiency changes that result in re‐spending of any monetary savings on some other goods and services that, again, require energy for production (Freire‐González, 2017; Sorrell et al., 2009). In this line of thinking, indirect rebound effects are intrinsically linked to the direct rebound effect—but can also occur as a direct consequence of behavioural changes, for example, just through more eco‐friendly behaviour, that is, not linked to energy efficiency (Freire‐González, 2017). Table 1 presents a set of behaviours depicting how this may influence climate change, the kind of impact this behaviour implies as well as the scope of potential GHG reduction.

**TABLE 1:** How some household‐level behaviours may influence climate change.

	Examplatory behaviour	Influence on climate change	Impact	Alternative behaviour	Reduction potential/scale of intervention
1	Flying frequently	Significantly increases aviation‐related CO_2_ and other greenhouse gas emissions	(−)	Low‐carbon travel options, virtual meetings	Large emission reduction potential; immediate, tangible benefit if addressed
2	Carnivore diet	High methane emissions from livestock and deforestation for animal grazing and feed	(+)	Adopting plant‐based diet	Large emission reduction potential; immediate, tangible benefit if addressed
3	Excessive home energy use (air conditioning/heater etc.)	Raises energy demand, often from fossil fuels, increasing GHG emissions	(−)	Shading, better insulation, solar balcony power plants	Large emission reduction potential
4	Wasting food	Leads to higher methane emissions from landfills and wasted agricultural resources	(−)	Conserving, composting, mindful shopping	Modest emission reduction potential; immediate, tangible benefit if addressed
5	Landfilling and incineration	Lowers landfill waste, reduces methane emissions, and decreases resource extraction	(+)	‘Reduce, reuse and recycle’, composting	Medium emission reduction potential, 5% increase in recycling of low value waste reduces GHG emissions by 10%; composting resulted in 39 to 84% lower CH_4_ emissions than landfilling
6	Fast fashion consumption	Increases carbon footprint due to textile production, shipping and landfill waste	(−)	Slow fashion, i.e. conscious sustainable purchasing, second hand	High emission reduction potential due to lower carbon‐intensive traditional fashion clothing production processes
7	Using fossil‐fuel‐powered vehicles	Increases CO_2_ emissions, contributing to global warming	(−)		Large emission reduction potential, EVs with 73% less carbon emissions during driving than conventional vehicles

*Source*: Authors, amended with findings from Guan et al. (2025), Pérez et al. (2023), Li et al. (2024), Li and Chu (2025), IEA (2025) and Du et al. (2025).

With its influence on the health of humans, climate change has direct impacts such as that of rising temperatures that impact sleep, (Minor et al., 2022), as well as indirect implications such as climate anxiety and heightened stress from climate‐related disasters (Gago et al., 2024; Stolz, 2025). To be more resilient to the health impacts of climate change, people should therefore engage in specific health behaviours, that is, climate‐adaptation related action. Simple changes here can safeguard human health and promote overall health. For instance, during heatwaves, staying hydrated and dressing appropriately can mitigate personal risk of heat‐related illness (Ollie et al., 2021). On a larger scale, that is, societal scale, adopting eco‐friendly behaviour, like eating more plant‐based food, making changes to reduce food waste and choosing to walk or cycle, also benefits public health and builds your capacity to cope with climate change.

**FIGURE 1:**
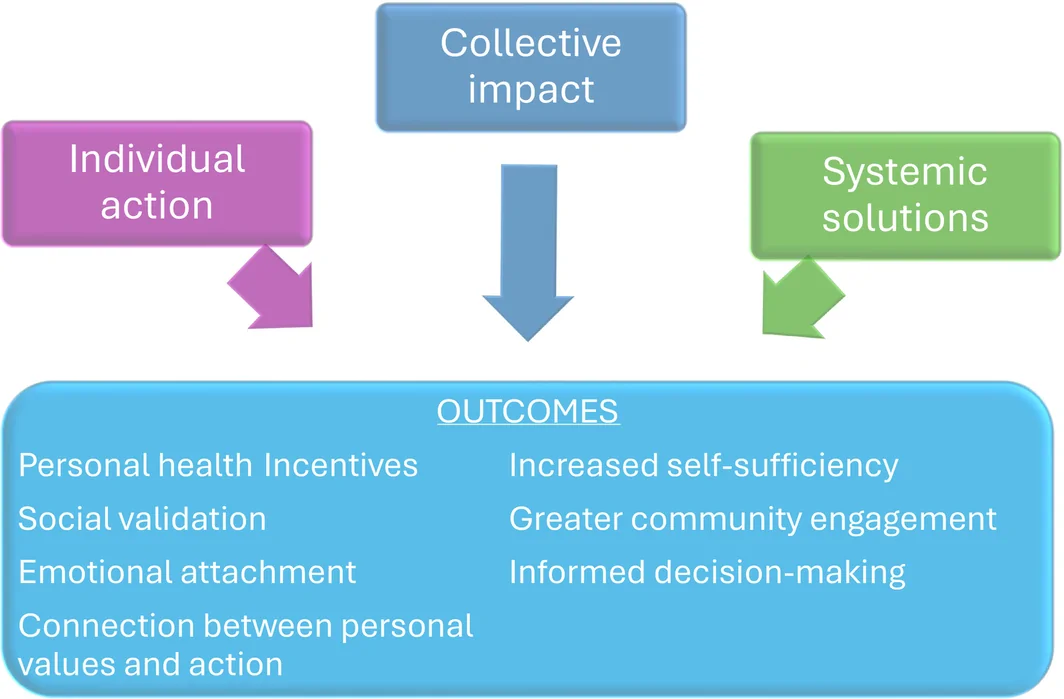
Some key drivers of climate and health behaviours. Source: Authors.

Integrating climate change and planetary health into health disciplines becomes paramount as accelerated climate change is driven by our continued unsustainable behaviours, and, at the same time, health is increasingly undermined by progressing climate change. Health psychology, in particular, should address climate change and planetary health issues better (Chevance et al., 2022). Bridging health psychology and climate research, and also understanding the psychological aspects of climate change and health, is important. Here, health psychologists can play a key role in encouraging climate action in line with SDG13 since many key mitigation behaviours have health co‐benefits and are influenced by similar processes as health behaviours (Papies et al., 2024). Moreover, by promoting mental health and well‐being, health psychologists simultaneously address an interlinked SDG, that is, SDG3's target on health and well‐being. The reasons why individuals act on climate change are complex and shaped by various health, economic and environmental factors. While traditional metrics of knowledge do not strongly predict action, beliefs of self‐efficacy and social pressure are more reliable indicators of environmental behaviour (Singh et al., 2024). Behaviour change models such as the BUCkEt model (Fiske, 2018) help to show how motivation can be linked to core social values. This model emphasises the role of social motivations, such as belonging and a sense of control, in driving environmentally friendly actions. Still, it is important to tackle the psychological barriers that prevent people from aligning their beliefs with climate change actions (Brick et al., 2021). Figure 1 presents an overview of some key drivers to climate and health behaviours and related outcome variables, with self‐sufficiency referring to one's individual assessment of functioning across multiple and specific aspects of well‐being in a potentially adverse or worsening climatic environment (Fluit et al., 2023).

Strategies that promote behaviour change to drive climate action are possible, if the complex challenges faced—and their ramifications—are understood. Health considerations should be embedded into climate change policies and strategies to minimise health risks and ensure that health benefits are maximised. Furthermore, a robust health system is important to prepare communities to meet the health threats of climate change, combating heat‐related illnesses, air quality problems and infections.

Aligning climate action to public health objectives means conducting comprehensive health impact assessments that consider the various factors affecting population health in a changing climate. Accordingly, undertaking lifestyle changes is essential to minimising health risks from climate change, including behaviour that may reduce carbon emissions at the same time as building resilience to environmental‐based health issues. The integration of health psychology, encompassing behavioural, psychosocial and biomedical sciences and climate science is also pivotal in developing integrated strategies that impact health outcomes, while incorporating the social determinants of health. Such feedback loops between climate change and behaviours are critical to designing effective interventions that prioritise health equity, even as the challenges presented by a changing climate differ greatly. Health psychology may help in encouraging habits that promote health and may reduce negative climate impacts. Clear, actionable messages can be very effective in appealing to societal expectations to indicate how many people support climate solutions. A health‐oriented message focusing on solutions can also persuade sceptics or disinterested audiences better (Thier et al., 2025). A focus on immediate health effects of action on climate, for example, improved heat resilience, reduced risk of cardiovascular diseases—rather than only focusing on long‐term outcomes or risks—could be vital in motivating people to pro‐environmental actions (Peters et al., 2022).

Motivation in driving action on climate change is an intricate network of interconnections through health and environmental context. Although evidence suggests that knowledge and experience correlate weakly to adaptive behaviours, the perceived self‐efficacy concept and social norms have been shown to be stronger predictors of pro‐environmental behaviours (Acquadro Maran et al., 2023; Plohl et al., 2026; Pradhananga & Davenport, 2022; Vrselja et al., 2024). Health behaviour change models (HBCMs) may be useful in highlighting how perceptions of vulnerability, severity and self‐efficacy are related to the level of engagement with climate‐related initiatives (Brown et al., 2023). Incorporating health considerations into adaptation and mitigation measures for climate change is important to lower the risks of health consequences and enhance the beneficial effects.

Through acknowledging the connection between climate action and public health targets, state‐of‐the‐art health impact assessments today need to account for health risks as well as further stressors affecting a population's well‐being and thus must focus on both to respond accordingly to current policy.

## HOW BEHAVIOURAL CHANGE CAN HELP ADDRESS THE HEALTH IMPACTS OF CLIMATE CHANGE TO ACHIEVE SDG 13

To facilitate impactful behavioural change, it is important to identify the specific type of underlying climate action that results in health co‐benefits. Mitigation initiatives range from individual‐level decisions—for example, selecting sustainable, low‐emission transport options resulting in reduced ground‐level air pollution—to broader community‐centred efforts aimed at improving urban design and local air quality (Javaid et al., 2020; Thurston & Bell, 2020). Adaptation initiatives may range from increased awareness of the role of individual hydration in hotter climatic environments (Epstein & Moran, 2019) to seizing urban green space cooling effects (Aram et al., 2019) to smart shading building design for thermal comfort also during higher temperatures (Heidarzadeh et al., 2025).

### Role of psychological barriers

Despite the clear need for behavioural change, it is also known that a range of psychological barriers can hinder people from engaging in environmental behaviour (see Table 2). Moreover, uncertainty, distrust of information sources and competing life priorities frequently restrain people from engaging in pro‐environmental behaviour. Gifford (2011) published the finding that psychological barriers, including limited cognition about the problem and risk perception, reduce active adoption of climate‐friendly practices especially energy conservation and sustainable food choices (Vieira et al., 2023). Addressing these psychological challenges is the key to converting intentions into action and fostering a proactive climate engagement culture.

**TABLE 2:** Selected psychological barriers preventing individuals from acting on their environmental convictions.

Psychological barrier	Description
Cognitive dissonance	When a person's actions do not align with their environmental beliefs, they may rationalise their behaviour to reduce discomfort rather than change it.
Moral licensing	After performing one pro‐environmental action (e.g. recycling), individuals may feel justified in engaging in less sustainable behaviours later.
Perceived behavioural control	People may believe their individual actions will not make a meaningful difference, leading to inaction.
Social norms and conformity	If sustainable behaviour is not the norm in one's social group, individuals may hesitate to act differently to avoid standing out.
Psychological distance	Environmental issues (e.g. climate change) may feel abstract, distant in time or space, reducing urgency to act.
Optimism bias	The belief that negative environmental outcomes will not personally affect them, leading to complacency.
Habit inertia	Established routines (e.g. driving instead of biking) are hard to break, even when individuals know they should change.
Defensive avoidance	Overwhelming environmental problems may lead people to avoid thinking about them altogether.
Confirmation bias	People may seek information that aligns with their existing (often unsustainable) behaviours and ignore contradictory evidence.
Just‐world hypothesis	The belief that the world is inherently fair may lead individuals to assume environmental problems will resolve themselves without their intervention.

*Source*: Authors.

As discussed before, increasing the motivation for actively engaging with climate change adaptation and mitigation action is a key lever for overcoming such psychological barriers. It may be very beneficial to appreciate the co‐benefits from enhancing public health by delivering climate‐adaptation strategies. Interventions targeting local environments—such as the creation of cooling centres in urban settings—should also encourage health benefits to include increased physical activity and reduced risks associated with heat‐related conditions. But due caution is required to prevent unintended side‐effects that exacerbate current health inequalities and in particular for underserved individuals who tend to be severely disadvantaged by health, socio‐economic status and access to necessary resources (Cheng & Berry, 2012).

Health equity issues need thus to be addressed as well when looking at the interlinkages of climate change, health and behaviour, as systemic inequities across systems and societies can constrain behavioural change. In the light of progressing climate change, which may exacerbate existing challenges like sufficient food production to counteract malnutrition, more equitable and resilient systems could ensure improved access to resources, for example, enabling healthier diets or reduce the risk of illness on society (Fanzo et al., 2022). Inclusive strategies incorporating distinctive at‐risk, that is, vulnerable, groups can also help achieve fairer health outcomes yet Vromans et al. (2023) question in their meta‐review that providing personalised health risk information leads to strong or sustained impacts on health‐related behaviour. The following Table 2 depicts selected psychological barriers that may hinder individual climate action.

### Factors influencing climate action

Understanding individual motivations for climate action reveals a complex interplay of health, economic and environmental factors. Research indicates that motives such as health benefits often drive the adoption of sustainable food practices (Baur et al., 2022; Chen et al., 2024), while economic incentives stimulate decisions related to sustainable transport (Andersson et al., 2020) and energy consumption (Słupik et al., 2021). It is important to consider these divergent motivations to inform the development of climate communication approaches:

- For tackling climate, messages promoting both the immediate human health rewards of sustainable choices and their larger social consequences are relevant for different segments of the population; therefore increasing participation (Brown et al., 2023).
- Education campaigns are an important enabler of behavioural changes within the principles of community health initiatives. Climate change education has been found to be an effective mechanism to promote better health behaviour change for specific demographic groups. The impact on health‐related behaviours is particularly strong for vulnerable populations, such as children, who often experience substantial challenges to change, primarily because of socio‐economic reasons due to low‐income children and other socio‐economic circumstances. Climate change literacy programmes also educate people on more healthful and sustainable decisions that may translate into improved individual health status and lead to better health outcomes, in support of a climate‐adaptation model that seeks to increase future resilience (Badawy et al., 2023).
- Community engagement is a key factor for successful climate change adaptation events. Building on the characteristics of participatory research in the community, this framework will enable local populations to share health resources in relation to the health effects of climate impacts and therefore guide intervention in a manner that is responsive to local circumstances and populations. This community is useful not only for improving the relevance and efficacy of health strategies but also for nurturing social networks that act as crucial resources of support in the face of climatic adversities (Black et al., 2024).
- Emerging technologies are promising means to encourage sustainable behaviours and public health engagement. Numerous digital interventions such as gamification and immersive technologies have been found to promote the uptake of behaviours, such as energy saving and pollution reduction (Mosca et al., 2024). From the technological progression of these devices, we can further personal engagement as well as boost community‐based efforts to effectively meet climate and health challenges.

In summary, integrating insights from behavioural change into public health responses is fundamental both in terms of addressing health‐related implications of global warming and achieving the SDG 13 target. Building resilient communities and improving public health can be achieved by incentivising individual good practices that improve both personal health and help save our environment. Active and mobilising initiatives, grounded in local contexts, that utilise alternative modes of communication will also be essential in effecting substantive change.

Community participation is the very basis for positive climate adaptation and health‐related research. To do this, participatory research needs to engage local people and use their knowledge to respond to local environmental issues faced by marginalised populations. Such an inclusive approach helps improve the transferability of interventions and the fitness and applicability of health‐based research to continually transform the changing effects of climate change. These collaborations to implement community‐based participatory research or citizen science help to deepen knowledge of the intersection between climate impacts and health to form relationships that will guide adaptation and mitigation efforts (Black et al., 2024).

## CONCLUSIONS

Interventions aimed at promoting sustainable behaviours, that is, reducing harmful activities and inducing pro‐environmental action as well as adaptive capacities, can be diverse in targeting changes in culture, feedback loops and technological development, for instance, on community level. Also, an awareness about the driving forces towards climate‐resilient lifestyle changes might also make large contributions in responding to climate‐related risks to human health. Cross‐disciplinary perspectives between psychologists and social scientists can also foster a better understanding of the psychological processes that underpin climate change‐related behaviours, to provide an opportunity to combine public health measures with sustainable practice (Nielsen et al., 2020).

Further investigating the health benefits of adapting to climate change may also highlight the co‐benefits of climate‐adaptation strategies. Building a robust public health infrastructure enhances its capacities for health services and reduces the risks associated with natural hazards. To align climate actions to public health goals, comprehensive health impact assessments need to be conducted that encompass a number of health risks and stressors that affect the population as a whole (Cheng & Berry, 2012).

There is a strong need for effective communication strategies to inform the public and empower them on climate change/health as well as behavioural changes. Effective strategies to develop messages that are evidence‐based and tailored to varying populations should be implemented, thereby strengthening both knowledge dissemination and healthy behaviour promotion. Approaches should be especially based on drawing on sources of trust, activating social networks and promoting common norms and emotional appeals (Peters et al., 2022).

Finally, focusing on the health benefits of pro‐environmental action today and discussing the health threats presented by climate change may pave the way for improvements in this field. A holistic climate change response, however, needs to consider health concerns from a multisectoral perspective and engage stakeholders at all levels—from neighbourhood communities to international structures. Such an approach could allow multiple Sustainable Development Goals to be achieved and result in synergies between multiple sectors. Indeed, it is important to acknowledge the link between climate action, public health and sustainable development (Türkeş, 2024). A collective attitude to resilience to counteract climate change can shape the future of health and resilience in society.

## CONFLICT OF INTEREST STATEMENT

The authors declare no conflicts of interest.

## ETHICS STATEMENT

The authors confirm that the ethical policies of the journal have been adhered to. No ethical approval was required for this study as it is a review article containing no original clinical data or animal experimentation.

## Data Availability

No empirical data are used.
